# Comparative transcriptome analysis reveals potential fruiting body formation mechanisms in *Morchella importuna*

**DOI:** 10.1186/s13568-019-0831-4

**Published:** 2019-07-12

**Authors:** Haibo Hao, Jinjing Zhang, Hong Wang, Qian Wang, Mingjie Chen, Jiaxiang Juan, Zhiyong Feng, Hui Chen

**Affiliations:** 1 National Research Center for Edible Fungi Biotechnology and Engineering, Key Laboratory of Applied Mycological Resources and Utilization, Ministry of Agriculture, Shanghai Key Laboratory of Agricultural Genetics and Breeding, Institute of Edible Fungi, Shanghai Academy of Agricultural Sciences, 307 Room, No. 1000, Jinqi Road, FengXian District, Shanghai, 201403 China; 2 College of Life Science, Nanjing Agricultural University, No. 1, Weigang Road, XuanWu District, Nanjing, 210095 China

**Keywords:** *Morchella importuna*, Transcriptome, Carbohydrate-active enzymes, Mitochondria, Oxidoreductase, Heat shock protein

## Abstract

*Morchella importuna* has been artificially cultivated, but stable production remains difficult because its mechanisms of fruiting body formation are unclear. To investigate the fruiting body formation mechanisms, we sequenced the transcriptomes of *Morchella importuna* at the mycelial and young fruiting body stages. Among the 12,561 differentially expressed genes (DEGs), 9215 were upregulated, and 3346 were downregulated. DEG enrichment analysis showed that these genes were enriched in the “generation of precursor metabolites and energy”, “carbohydrate catabolic process”, and “oxidoreductase activity” Gene Ontology (GO) functional categories. Enzyme activity assay results indicated that the activity levels of CAZymes (carbohydrate-active enzymes), oxidoreductases (SOD (superoxide dismutase), CAT (catalase)) and mitochondrial complex (complex I, II, III) proteins were significantly increased from the mycelial stage to the young fruiting body stage. In addition, the genes encoding CAZymes, mitochondrial proteins, oxidoreductases and heat shock proteins had higher expression levels in the young fruiting body stage than in the mycelial stage, and the qRT-PCR results showed similar trends to the RNA-Seq results. In summary, these results suggest that carbohydrate catabolism and energy metabolism are significantly enhanced in the young fruiting body stage and that growth environment temperature changes affect the formation of fruiting bodies.

**Electronic supplementary material:**

The online version of this article (10.1186/s13568-019-0831-4) contains supplementary material, which is available to authorized users.

## Introduction

Morels, which are one of the most highly prized mushrooms with notable nutritional and medicinal value, belong to the ascomycetes (Liu et al. 2018a). Morels are rich in carbohydrates and proteins and contain several bioactive compounds, such as organic acids, phenolic compounds and tocopherols (Heleno et al. 2013). In addition, their bioactive compounds have been reported to possess immunoregulatory, antioxidant, antitumor, antibacterial and anti-inflammatory properties (Gursoy et al. 2009; He et al. 2012; Liu et al. 2017). The current phylogenetic analyses of morels mainly include the following three lineages: a basal monotypic lineage represented by *M. rufobrunnea* and two sister clades comprising black and yellow morels (Liu et al. 2017, 2018a). Many mushroom cultivators usually artificially cultivate the black varieties, such as *M. sextelata*, *M. septimelata* and *M. importuna*, and *M. importuna* accounts for more than 80–90% of the cultivated area in China (Kuo et al. 2012; Liu et al. 2018a). Notably, exogenous nutrition bags must be placed on the culture medium to provide sufficient nutrients for the development of the mycelium into the fruiting body (Liu et al. 2017, 2018a). The application of exogenous nutrition is the most important breakthrough in the field of morel cultivation, but its mechanism of action remains unclear (Liu et al. 2018a).

It has been reported that sclerotium formation by morels plays an important role in fruiting body formation (Ower 1982; Liu et al. 2017). The sclerotium may be a nutrient storage organ used while awaiting favorable conditions for fruiting body production (He et al. 2018). In addition, the growth substrates and their nutritional composition affect both mycelial characteristics and sclerotium formation (Liu et al. 2017). A comprehensive transcriptome analysis of *M. importuna* has suggested that the catabolism of carbohydrates occurs in the mycelial growth stage and that the sclerotial morphogenesis stage mainly involves the anabolism of energy-rich substances (Liu et al. 2019). Morels usually obtain nutrition for growth and reproduction via lignocellulose degradation (Liu et al. 2017). Three different endoglucanases (Endo I, Endo II and Endo III) and three different cellobiohydrolases (Exo I, Exo II and Exo III) have been purified from *M. conica* (Cavazzoni and Manzoni 1994). On the one hand, in *M. crassipes*, ligninolytic activity was affected by different carbon and nitrogen sources and inducers. On the other hand, laccase activity also plays an important role in sclerotium formation (Liu et al. 2017).

Unlike the cultivation methods of many other mushrooms, solid morel spawn is sown directly into cropland. The morel mycelia grow at soil temperatures of 2–20 °C and soil humidity of 50–70%, often requiring overwintering (maintenance of a relatively cold environment for 20 to 30 days) (Liu et al. 2017, 2018a). When the temperature increases to 6–8 °C, the air humidity reaches 85–90%, and the soil humidity reaches 65–75%, the primordium is stimulated to differentiate into the fruiting body (Liu et al. 2017, 2018a). This pattern indicates that temperature change and humidity are key factors in the field cultivation of morels.

The previous transcriptome analysis was conducted at the stages of mycelium, initial sclerotium and mature sclerotium, and it revealed some of the molecular mechanisms of mycelium growth (Liu et al. 2019). However, there is currently no research on the transcriptomes of the mycelia and young fruiting bodies of *M. importuna*, and the molecular mechanism of *M. importuna* fruiting body formation remains unclear.

In this report, we used liquid spawn of *M. importuna* to sow into cropland and achieved successful artificial cultivation of *M. importuna*. The artificial cultivation of *M. importuna* is affected by many factors, such as temperature, humidity and light (Liu et al. 2018a). Thus, to better understand the molecular mechanism of fruiting body formation, the transcriptomes of the mycelia and young fruiting bodies of *M. importuna* were analyzed. The aim of the current study was to examine the changes in gene expression from the mycelium stage to the fruiting body stage and thus reveal the possible mechanism of fruiting body formation. We also investigated the activities of CAZymes, oxidoreductases (SOD, CAT) and mitochondrial complex proteins (complex I, II, and III); the activities of the key enzymes involved in fruiting body formation and the expression levels of the genes encoding these enzymes were further studied. This transcriptomic information could increase our understanding of the molecular mechanisms of fruiting body formation and provide theoretical support for further improvement of artificial cultivation techniques.

## Materials and methods

### Fungal strain, growth conditions, and developmental stages

The *M. importuna* strain M-311 (CGMCC5.2201) (deposited in the China General Microbiological Culture Collection Center) was grown at 20 °C in potato dextrose agar medium for 5 days. Mycelial cultures were grown in liquid medium containing 2% glucose, 0.3% peptone, 0.5% soya bean meal, 0.1% MgSO_4_, and 0.15% KH_2_PO_4_ on a rotary shaker incubator at 150 rpm at 20 °C for 7 days. The mycelia were collected and washed with a large amount of distilled water. The cultured mycelia were further expanded and transferred to cropland (soil medium). The mycelia were grown in a spawn running process to allow mycelial maturation in soil medium at a temperature of 2–20 °C (December of the first year to January of the second year, 30–40 days). Then, a mycelial low-temperature care stage at 1–3 °C (30–40 days) was employed, and formation and growth of fruiting bodies occurred at 2–15 °C (20–30 days), after which the young fruiting bodies were collected (The diameter and height of the young fruiting bodies were 0.5–0.8 cm and 2–3 cm, respectively. The mushroom caps were dark brown, the ridges were raised, and pits had begun to appear. The mushroom stalks were white, and the bases were striated.). Samples from the mycelia and young fruiting bodies were frozen at − 80 °C for RNA extraction. Three biological replicates of the mycelial and young fruiting body stages were collected, and a total of six samples were used for the transcriptomic analysis.

### RNA extraction, library preparation and Illumina HiSeq sequencing

Total RNA was extracted from the tissue using TRIzol^®^ Reagent according to the manufacturer’s instructions (Invitrogen), and genomic DNA was removed using DNase I (Takara). Then, RNA quality was determined using a 2100 Bioanalyzer (Agilent) and quantified using an ND-2000 (NanoDrop Technologies). A high-quality RNA sample (OD260/280 = 1.8–2.2, OD260/230 ≥ 2.0, RIN ≥ 6.5, 28S:18S ≥ 1.0, > 10 μg) was used to construct the sequencing library.

RNA-Seq transcriptome libraries were prepared with the TruSeq™ RNA sample preparation kit from Illumina (San Diego, CA) using 1 μg of total RNA. Briefly, messenger RNA was isolated by poly(A) selection with oligo(dT) beads and fragmented using fragmentation buffer. Then, cDNA synthesis, end repair, A-base addition and ligation of the Illumina indexed adaptors were performed according to Illumina’s protocol. The libraries were then size-selected for cDNA target fragments with a length of 200–300 bp on 2% Low Range Ultra Agarose followed by PCR amplification using Phusion DNA polymerase (NEB) for 15 PCR cycles. After quantification by TBS380, paired-end libraries were sequenced by Shanghai Biozeron Biotechnology Co., Ltd. (Shanghai, China) on an Illumina HiSeq with PE 2 × 150 bp read length. All reads were deposited in the National Center for Biotechnology Information (NCBI) database under accession number SRP192732.

### De novo assembly and annotation of *M. importuna* transcriptome

The raw paired-end reads were trimmed and quality controlled by Trimmomatic with the default parameters (http://www.usadellab.org/cms/uploads/supplementary/Trimmomatic). Then, clean data from all samples were used to perform RNA de novo assembly with Trinity (http://trinityrnaseq.sourceforge.net/) (Grabherr et al. 2011). All the assembled transcripts were searched against the NCBI protein nonredundant (NR), String, and Kyoto Encyclopedia of Genes and Genomes (KEGG) databases using BLASTX to identify the proteins that had the highest sequence similarity with the given transcripts and retrieve their functional annotations; a typical cutoff E-value of less than 1.0 × 10^−5^ was used. The BLAST2GO (http://www.blast2go.com/b2ghome) (Conesa et al. 2005) program was used to obtain the Gene Ontology (GO) annotations of the unique assembled transcripts to assess their functional enrichment in the biological process, molecular function and cellular component categories. Metabolic pathway analysis was performed using KEGG (http://www.genome.jp/kegg/) (Goto 2000).

### Differential expression and functional enrichment analyses

To identify DEGs between the two sample sets, the expression level of each transcript was calculated according to the reads per kilobase of exon per million mapped reads (RPKM) method. RSEM (http://deweylab.biostat.wisc.edu/rsem/) (Li and Dewey 2011) was used to quantify gene and isoform abundances. The R statistical package software EdgeR (Empirical analysis of Digital Gene Expression in R, http://www.bioconductor.org/packages/2.12/bioc/html/edgeR.html) (Robinson et al. 2010) was utilized for differential expression analysis. The p-value for multiple tests was determined by the value of the false discovery rate (FDR). We used ‘FDR ≤ 0.05 and |log2FC | ≥ 2’ as the threshold to judge the significance of gene expression differences. In addition, functional enrichment analyses, including GO and KEGG analyses, were performed to determine which DEGs were significantly enriched in GO terms and metabolic pathways at a Bonferroni-corrected P-value of ≤ 0.05 based on the whole-transcriptome background. GO functional enrichment and KEGG pathway analysis were carried out by Goatools (https://github.com/tanghaibao/Goatools) and KOBAS (http://kobas.cbi.pku.edu.cn/home.do) (Xie et al. 2011).

### CAZyme, mitochondrial protein, oxidoreductase and heat shock protein annotation

The annotation of unigenes from the *M. importuna* transcriptome related to CAZymes, mitochondrial proteins, oxidoreductases and heat shock proteins was performed via several common databases (the Carbohydrate-Active Enzymes (CAZy) database, the NCBI NR database, the Swiss-Prot protein (SWSS) database, the KEGG pathway database, GO functional databases and the Cluster of Orthologous Groups (COG) database), with a cutoff E-value of 10^−5^.

### Enzyme assay

Samples of *M. importuna* were ground into a fine powder in liquid nitrogen. Then, the samples (0.2 g) were homogenized in 1.8 mL of normal saline and centrifuged at 8000×*g* for 10 min. The supernatant was used for measurements of endo-1,4-beta glucanase, exo-1,4-beta glucanase, beta-glucosidase, beta-glucuronidase, SOD and CAT activity with the corresponding assay kits (Comin Biotechnology, Suzhou, China).

Endo-1,4-beta glucanase activity was measured according to the instructions of the assay kit. One unit of endo-1,4-beta glucanase was defined as the amount of enzyme that decomposed sodium carboxymethyl cellulose to produce 1 μg of glucose, as monitored at 540 nm. Exo-1,4-beta glucanase activity was measured according to the instructions of the assay kit. One unit of exo-1,4-beta glucanase was defined as the amount of enzyme that decomposed microcrystalline cellulose to produce 1 μg of glucose, as monitored at 540 nm. Beta-glucosidase activity was measured according to the instructions of the assay kit. One unit of beta-glucosidase was defined as the amount of enzyme that decomposed 4′-nitrophenyl-beta-d-glucopyranoside to produce 1 nmol of 4-nitrophenol, as monitored at 400 nm. Beta-glucuronidase activity was measured according to the instructions of the assay kit. One unit of beta-glucuronidase was defined as the amount of enzyme that decomposed phenol-β-d-glucuronic acid to produce 1 μmol of phenolphthalein, as monitored at 540 nm. CAT activity was measured according to the instructions of the assay kit. One unit of CAT was defined as the amount of enzyme that decomposed 1 μmol of H_2_O_2_, as monitored at 240 nm. SOD activity was measured according to the instructions of the assay kit. One unit of SOD was defined as the amount of SOD that inhibited 50% of hydroxylamine oxidation per gram of tissue in 1 mL of solution, as monitored at 550 nm.

The activity levels of mitochondrial complexes I, II and III in the samples were measured using assay kits (Comin Biotechnology, Suzhou, China) utilizing previously described methods (Liu et al. 2018c).

### Quantitative real-time PCR (qRT-PCR) validation

Total RNA was extracted from the two sample sets—mycelium and young fruiting body—by using a TRIzol Plus RNA Purification Kit. Then, approximately 2 μg of total RNA was reverse-transcribed to cDNA by M-MLV reverse transcriptase (Takara) using oligo(dT) primers. The unigenes of interest were subjected to quantitative real-time PCR (qRT-PCR) analysis. Additionally, the gene encoding the 14-3-3 protein was selected as the internal reference gene; this gene has been evaluated as a stable housekeeping reference gene in morels (Zhang et al. 2018). All primer sequences are shown in Additional file 1: Table S1. Amplification was carried out under the conditions previously described (Zhang et al. 2015). Relative gene expression was analyzed using the 2^−ΔΔCt^ method described by (Livak and Schmittgen 2001).

### Statistical analysis

All the experimental values are shown as the means ± standard deviations (SDs) of three independent experiments with three replicates each, and the data and graphs were processed using GraphPad Prism 6.0. Differences among treatments were analyzed by one-way analysis of variance (ANOVA) combined with Duncan’s multiple range test at a probability of P < 0.05.

## Results

### Illumina sequencing, assembly and functional annotation of the *M. importuna* transcriptome

**Fig. 1:**
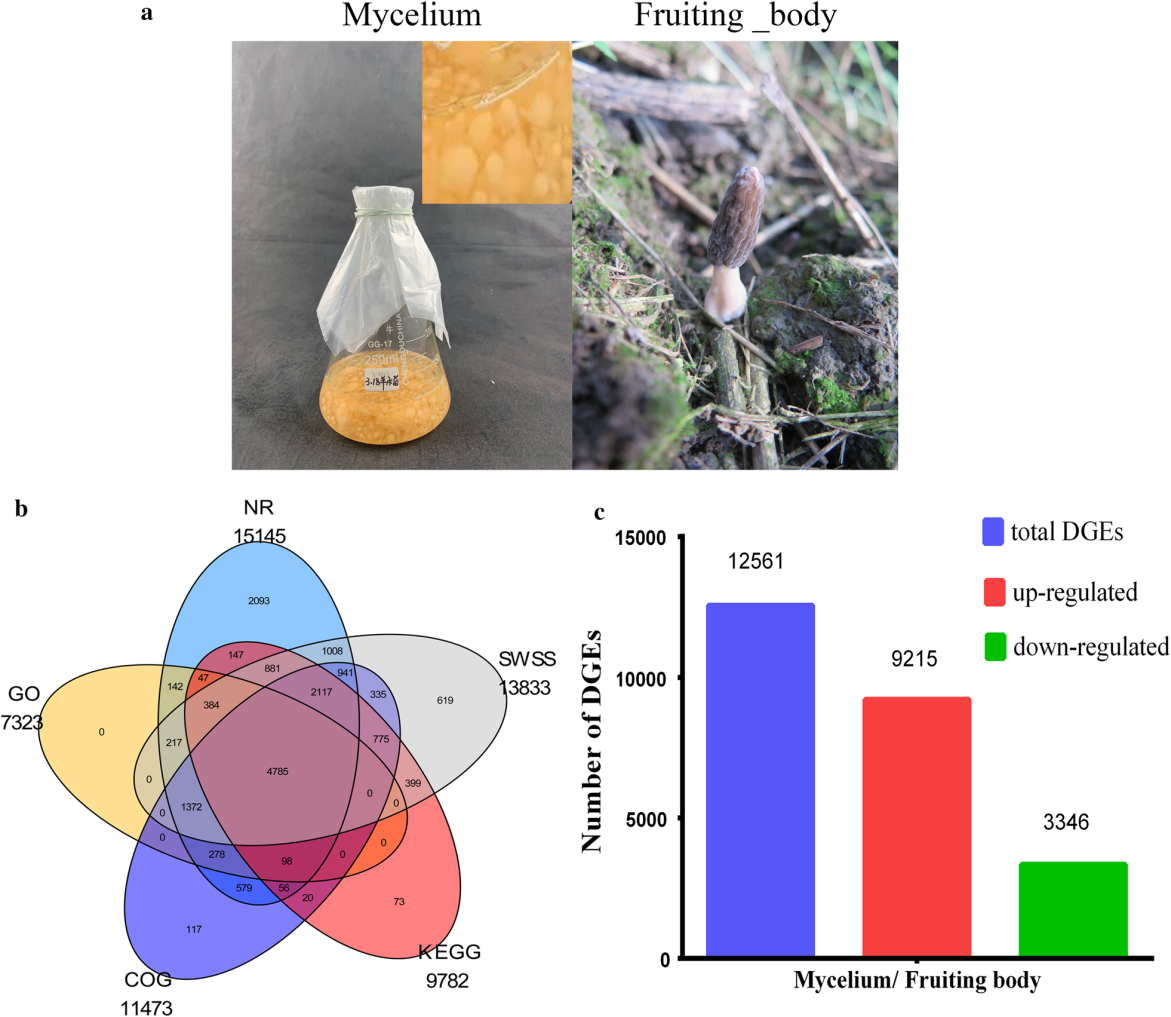
Summary of transcriptome annotation and differentially expressed genes (DEGs) between the *M. importuna* mycelium and fruiting body. **a** Representative pictures of a liquid shake flask culture of *M. importuna* mycelium at 7 days (left) and an artificially cultivated young fruiting body at 80 days (right). **b** A total of 33,269 unigenes, among which 15,145 (45.52%) were annotated in the NR database, 13,833 (41.58%) were annotated in the SWSS database, 11,473 (34.49%) were annotated in the COG database, 7323 (22.01%) were annotated in the GO database, and 9782 (29.4%) were annotated in the KEGG database. **c** The histogram shows the expression levels of 12,561 DEGs in the young fruiting body compared to the mycelium. The blue areas represent the total DEGs, the red areas represent DEGs that showed upregulated expression, and the green areas represent DEGs that showed downregulated expression

To obtain an overview of *M. importuna* gene expression during the mycelial and young fruiting body stages (Fig. 1a), cDNA samples were used for de novo transcriptome sequencing and sequence assembly. After sequencing, over 67.4 million raw reads and 101.15 billion raw bases were obtained in a single sequencing run (Table 1). Furthermore, clean data were obtained by removing reads with fewer than 70 bp and by adaptor trimming. Approximately 32.36 million and 26.19 million clean reads for which the Q30 value was over 96% were obtained from the mycelium and young fruiting body, respectively (Table 1). The obtained RNA sequences were assembled by using the sequence clustering software Trinity; 51,389 transcripts and 33,269 unigenes were generated. The longest and shortest unigenes were 42,312 and 351 bp, respectively. In addition, the average length of the unigenes was 1140.08 bp, with an N50 length of 1868 bp (Table 2).

**Table 1:** Read and base numbers for the *M. importuna* transcriptome

Samples	Mycelium	Fruiting body	Total
Raw bases	5,475,478,200	4,640,038,100	10,115,516,300
Raw reads	36,503,188	30,933,588	67,436,776
Clean bases	4,790,106,381	3,866,026,340	8,656,132,721
Clean reads	32,364,361	26,188,212	58,552,573
Mapped reads	24,868,848	18,809,366	43,678,214
Mapped ratio	76.73%	71.80%	–

**Table 2:** Results of the *de novo* transcriptome assembly of *M. importuna*

Type	Mycelium	Fruiting body	Total
Number of contigs	32,041	45,393	51,389
Average contig length (bp)	1517.28	1304	1257.04
Number of unigenes	21,335	30,051	33,269
Average unigene length (bp)	1429.91	1189.95	1140.08
Longest unigene (bp)	42,312	42,312	42,312
Shortest unigene (bp)	351	351	351
N50 (bp)	2304	1971	1868

The total number of open reading frames (ORFs) was approximately 51,386, of which 12,006 (56.27%) unigenes were predicted ORFs in the mycelium and 18,471 (61.46%) were predicted ORFs in the young fruiting body (Additional file 1: Table S2). The predicted protein sequences of the unigenes were annotated using BLASTX against the NR, STRING gene, GO, COG, KEGG, and SWSS databases with an E-value of 10^−5^; 17,483 (52.55%) predicted protein sequences were annotated as known unigenes (Fig. 1b).

Of the unigenes, 15,145 (45.52%) were annotated in the NR database. The distribution of the top 10 species by unique species hits in the NR annotation is shown in Additional file 1: Table S3. The NR annotation shows that the greatest numbers (3172 and 2663) of genes from *Tuber aestivum* and *Tuber melanosporum*, respectively, were well matched to *M. importuna* transcripts. A total of 11,473 (34.49%) transcripts were assigned to 25 functional categories by COG classification (Additional file 1: Fig. S1). Furthermore, we used GO assignments to classify the 7323 transcripts into 51 functional groups (Additional file 1: Fig. S2). According to the COG classifications and GO functional categories, most of the transcripts were associated with the growth and development of *M. importuna*. In addition, 9782 unigenes were annotated in the KEGG database and mapped to 344 reference canonical KEGG pathways. The KEGG pathways were mainly divided into five branches (Cellular Processes, Environment Information Processing, Genetic Information Processing, Metabolism and Organismal Systems); the highest numbers of transcripts were involved in “Global and overview maps” (2704 transcripts), followed by “Translation” (1186 transcripts), “Carbohydrate metabolism” (1085 transcripts), “Amino acid metabolism” (818 transcripts), “Signal transduction” (773 transcripts) and “Energy metabolism” (716 transcripts) (Additional file 1: Fig. S3). The high transcript numbers indicated that these pathways were related to the growth and development of *M. importuna* and involved in mass protein synthesis, carbohydrate metabolism, energy metabolism and signal transduction in response to environmental changes.

### Identification, characterization and enrichment of DEGs

Based on DEG analysis of the transcriptomes of the mycelium and young fruiting body, a total of 12,561 unigenes were identified as DEGs, comprising 9215 upregulated unigenes and 3346 downregulated unigenes in the young fruiting body formation stage (Fig. 1c). Based on GO functional classification, the two samples’ DEGs were assigned to three categories (biological process, cellular component, and molecular function) and divided into 39 subcategories. The highest numbers of DEGs were enriched in the “metabolic process”, “cellular process”, “cell”, “cell part”, “catalytic activity” and “binding” terms in the three categories. The “locomotion”, “biological adhesion”, “biological phase”, “protein binding transcription factor activity” and “protein tag” subcategories were the only terms with upregulated genes. In addition, the largest percentages of upregulated genes were involved in the “growth” (84.21%), “reproductive process” (83.33%), “membrane-enclosed lumen” (86.79%), “macromolecular complex” (86.60%), “structural molecule activity” (90.00%) and “enzyme regulator activity” (83.33%) subcategories (Additional file 1: Table S4).

In the GO functional enrichment analysis of all DEGs, 497 DEGs were enriched in the three GO functional categories and in 66 subcategories (Additional file 1: Fig. S4). In the biological process category, 259 of the 497 DEGs were mainly enriched in “generation of precursor metabolites and energy” (65), “carbohydrate catabolic process” (51) and “polysaccharide catabolic process” (23). In the cell component category, 88 genes were enriched in 13 subcategories, with “extracellular region” (23), “fungal-type cell wall” (17) and “respiratory chain” (15) dominant among these subcategories. In the molecular function category, 150 genes were mainly enriched in the “oxidoreductase activity” (30) and “structural constituent of cytoskeleton” (24) terms (Additional file 1: Fig. S4). In the KEGG enrichment analysis of all DEGs, 30 of 326 KEGG pathways were enriched; Additional file 1: Fig. S5 shows those that were significantly enriched, mainly including “Galactose metabolism”, “Metabolism of xenobiotics by cytochrome P450” and “Carbohydrate digestion and absorption”.

The results of the GO and KEGG functional classification and enrichment analyses indicated that the DEGs were mainly related to carbohydrate metabolism, energy metabolism and oxidoreductase activity in the young fruiting body formation stage.

### Differentially expressed CAZyme, mitochondrial protein, oxidoreductase and heat shock protein genes in *M. importuna*

**Fig. 2:**
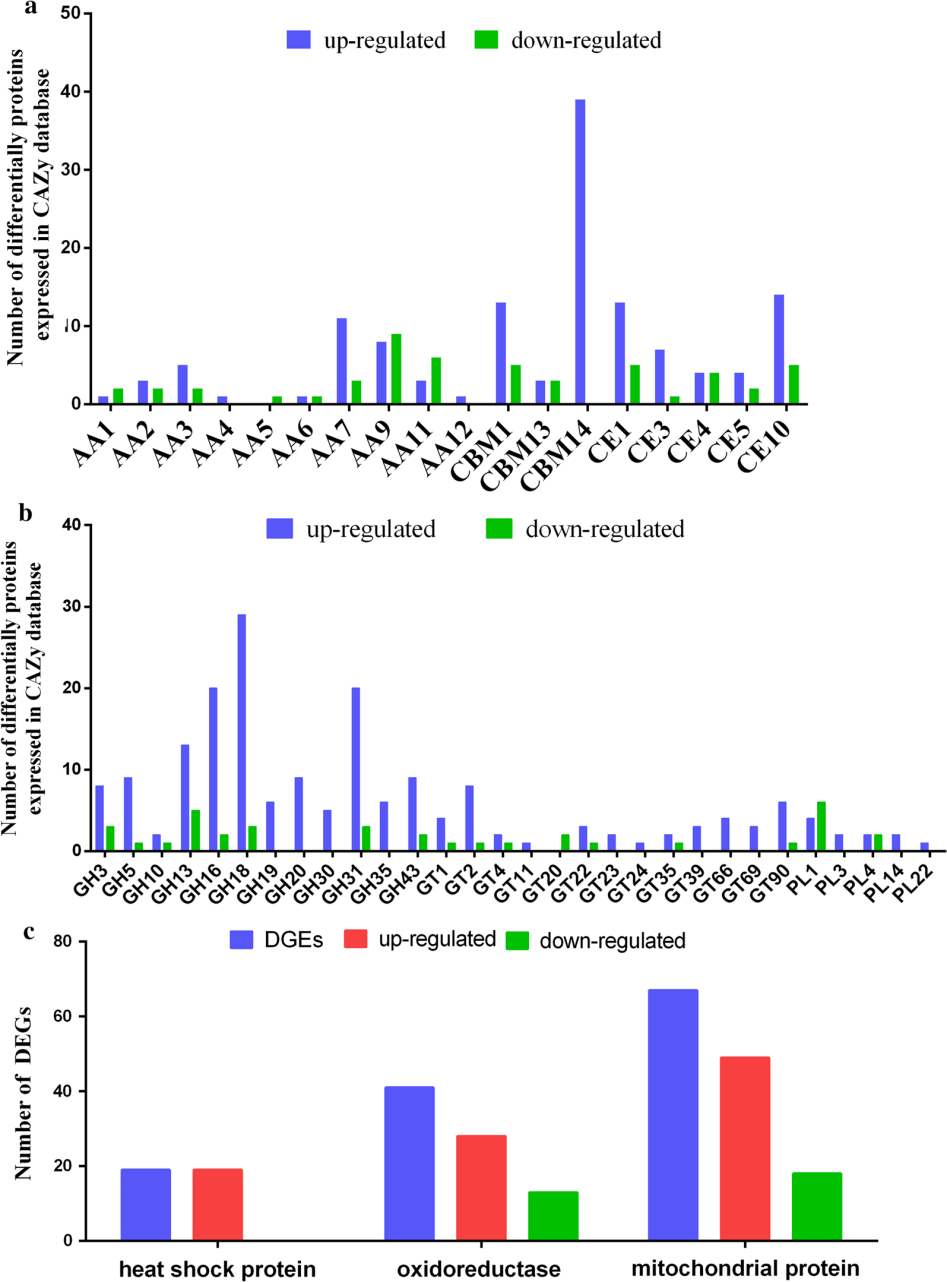
Histogram showing differentially expressed CAZyme, mitochondrial protein, oxidoreductase and heat shock protein genes. **a**, **b** The number of differentially expressed proteins in the CAZy database. The blue areas represent upregulated expression, and the green areas represent downregulated expression. **c** The number of mitochondrial, oxidoreductase and heat shock protein DEGs; the blue areas represent the total DEG number, the red areas represent genes with upregulated expression, and the green areas represent genes with downregulated expression

CAZymes are involved in carbohydrate metabolism and include six families of enzymes (auxiliary activities (AAs), glycosyl hydrolases (GHs), carbohydrate binding modules (CBMs), carbohydrate esterases (CEs), glycosyl transferases (GTs), and polysaccharide lyases (PLs)) (Andre et al. 2014). By comparing the whole transcriptome of *M. importuna* with the CAZy database, we identified 605 DEGs as CAZymes, among which 443 (71.57%) were upregulated and 172 (28.43%) were downregulated. Across the six families of CAZymes, the number of DEGs in the fruiting body formation stage was significantly higher than that in the mycelium stage; the main results are shown in Fig. 2a, b. Most of the oxidoreductase and mitochondrial complex DEGs were upregulated in the fruiting body formation stage, representing 68.29% and 73.13% of the DEGs in those respective categories (Fig. 2c). In addition, all heat shock protein DEGs were upregulated (Fig. 2c). Therefore, the mycelium forms fruiting bodies after environmental changes, which could be associated with upregulated expression of carbohydrate metabolism, energy metabolism, oxidoreductase and heat shock protein DEGs.

### Enzyme assay

**Fig. 3:**
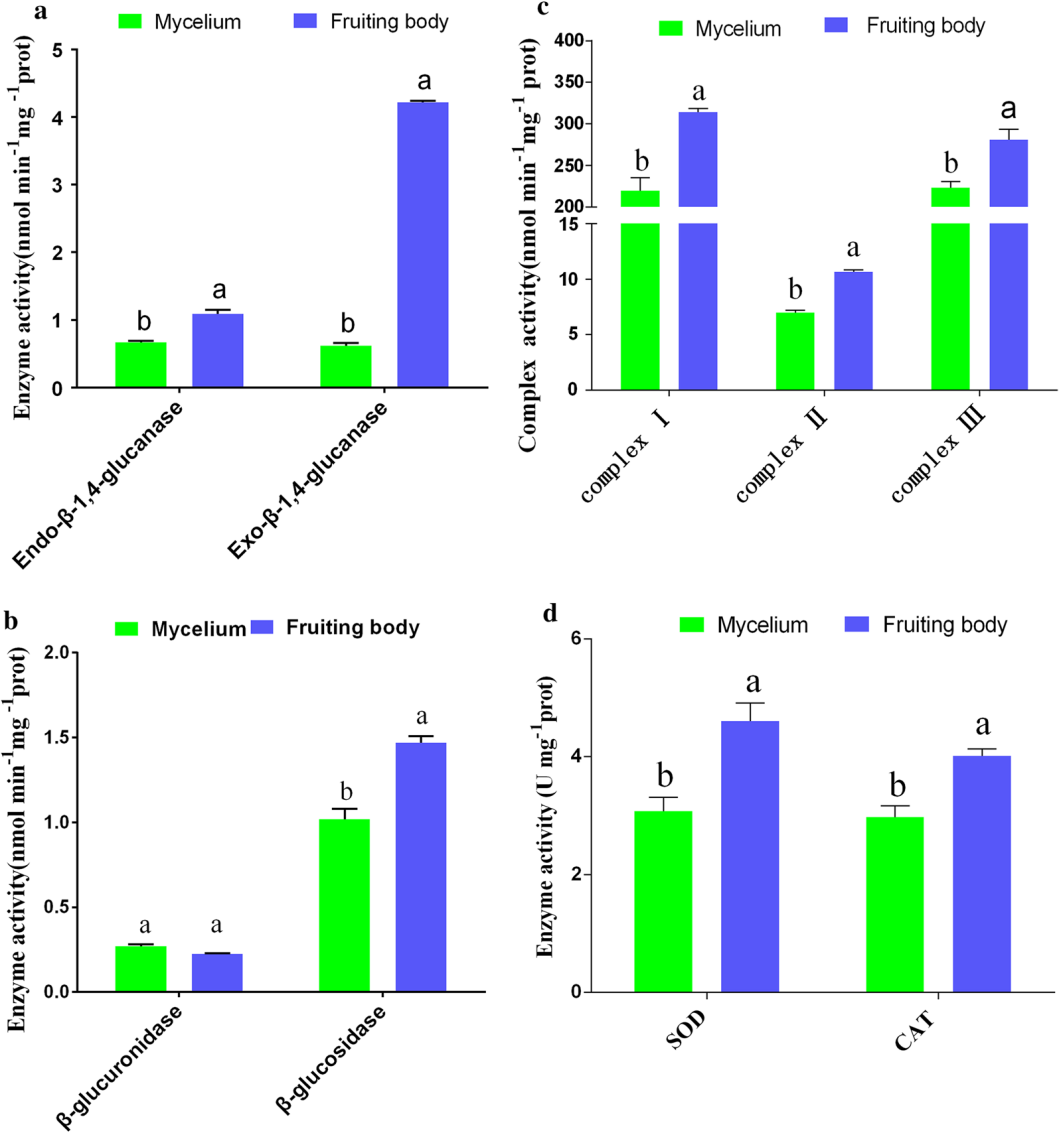
Histogram showing the enzyme activity of CAZymes, oxidoreductases and mitochondrial complex proteins. **a**, **b** Cellulase activities of exo-β-1,4-glucanase, endo-β-1,4-glucanase, β-glucosidase and β-glucuronidase. **c** Oxidoreductase activities of SOD and CAT. **d** Mitochondrial complex enzyme activities of complexes I, II, and III. All data are presented as the means ± SDs of three independent experiments. The different letters on the bars denote statistically significant differences compared with the enzyme activity in the mycelium according to multiple comparisons testing (P < 0.05)

To illustrate the changes in carbohydrate metabolism, the activities of oxidoreductase and ATP synthesis enzymes from the mycelial to the fruiting body formation stage, including endo-β-1,4-glucanase, exo-β-1,4-glucanase, β-glucuronidase, β-glucosidase, SOD, CAT and mitochondrial complex (complex I, II, and III), were measured in these two growth stages. As shown in Fig. 3a, b, the young fruiting body stage contained particularly high levels of cellulase (exo-β-1,4-glucanase, endo-β-1,4-glucanase and β-glucosidase) activity, with 6.77-fold, 1.63-fold and 1.44-fold higher activity, respectively, than in the mycelial stage. However, β-glucuronidase activity showed no significant difference (Fig. 3b). SOD and CAT activity levels were significantly higher in the young fruiting body stage than in the mycelial stage (Fig. 3d). In addition, mitochondrial complex I, complex II and complex III activity levels were increased 1.43-fold, 1.53-fold and 1.25-fold, respectively (Fig. 3c). These results indicate that the activity levels of CAZymes, SOD, CAT and mitochondrial complexes play important roles in the transition from the mycelium stage to the fruiting body stage under changing environmental conditions.

### Validation of CAZyme, mitochondrial complex, oxidoreductase and heat shock protein gene expression levels by qRT-PCR

**Fig. 4:**
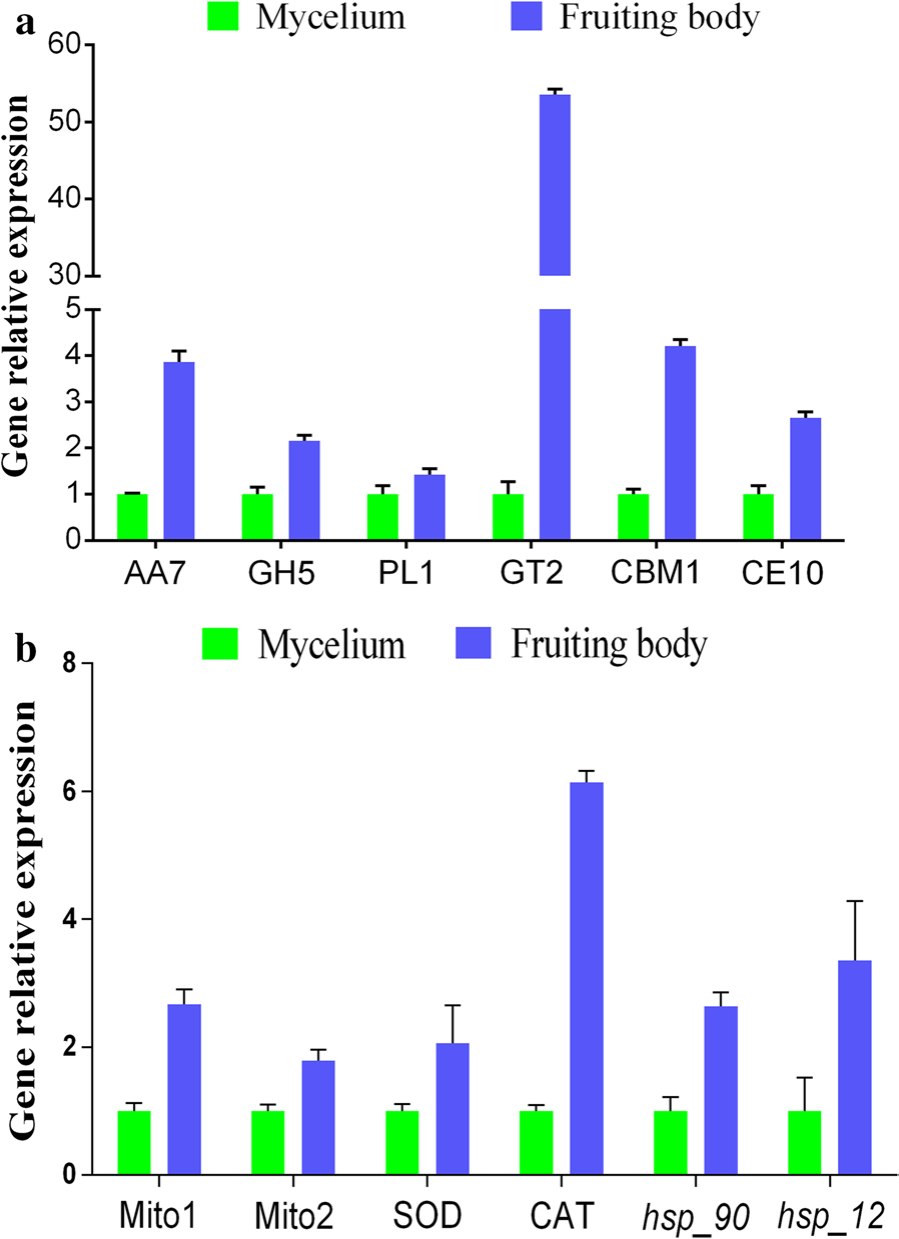
Validation of the gene expression levels of enzyme. **a** Six differentially expressed CAZymes (AA7, GH5, GT2, CBM1, CE10, and PL1). **b** Two differentially expressed oxidoreductases (SOD and CAT), two differentially expressed heat shock proteins (*hsp_12* and *hsp_90*), and two differentially expressed mitochondrial ATP synthases (Mito1: ATP synthase, Mito2: mitochondrial F1F0 ATP synthase). The error bars represent the means ± SDs of triplicate experiments

The expression levels of the genes involved in carbohydrate metabolism, energy metabolism, oxidoreductase activity and heat shock response were measured in the developmental stages by qRT-PCR. The genes detected comprised 6 CAZyme genes [AA7 (TRINITY_DN64230_c37_g1), GH5 (TRINITY_DN61551_c5_g1), GT2 (TRINITY_DN36423_c0_g1), CBM1 (TRINITY_DN63119_c0_g1), CE10 (TR INITY_DN58528_c15_g1), and PL1 (TRINITY_DN47004_c0_g1)], 2 heat shock protein genes [*hsp*-*_90* (TRINITY_DN10567_c0_g1) and *hsp*-*_12* (TRINITY_DN54 44_c0_g2)], 2 oxidoreductase genes [SOD (TRINITY_ DN5158 6_c0_g2) and CAT (TRINITY_DN6020_c0_g1)], and 2 mitochondrial ATP synthase genes [Mito1: ATP synthase (TRINITY_DN46199_c0_g1) and Mito2: mitochondrial F1F0 ATP synthase (TRINITY_DN5524_c0_g2)] in *M. importuna*. As shown in Fig. 4a, b, gene expression profiling of these DEGs using qRT-PCR revealed variation trends similar to those observed in the RNA-Seq results.

## Discussion

*M. importuna* is a rare edible and medicinal mushroom species (Liu et al. 2017). Due to the expansion of its cultivation areas and technical goals of high yield and production stability, the molecular mechanism of *M. importuna* growth and development has become a high-value research area (Liu et al. 2018a). Although *M. importuna* has been studied and cultivated for many years, the fruiting body formation mechanisms of *M. importuna* are still unclear. Based on previous cultivation experience, we successfully used the liquid spawn of *M. importuna* to artificially cultivate this morel species, and mycelia and newly differentiated young fruiting bodies were collected for transcriptome sequencing. Furthermore, we also analyzed the significantly differentially expressed proteins from the mycelium stage to the fruiting body stage and further analyzed the activity and relative gene expression of significantly differentially expressed proteins.

Recently, two monospores of *Morchella* with different mating types were first subjected to genome sequencing, which yielded de novo assembled haploid M04M24 and M04M26 genomes of 48.98 and 51.07 Mb, respectively (Liu et al. 2018d). Interestingly, we obtained raw transcriptome sequences of 36.5 and 30.9 Mb in the mycelium and fruiting stages, respectively (Table 1). These results suggest that the mycelium transcriptome data accounted for a larger proportion of the whole genome.

A previous *M. importuna* transcriptome study indicated that a total of 26,496 genes were annotated from the mycelial stage to the mature sclerotia (Liu et al. 2019). In this study, we obtained 33,269 genes from the mycelial stage to the fruiting stage, and *M. importuna* expresses a larger number of genes in the young fruiting body stage (Table 1). These results indicate that the formation of fruiting bodies requires more gene involvement. In addition, a previous study on the gene expression profiles of *Schizophyllum commune* indicated that genes involved in fatty acid metabolism were upregulated in the fruiting body stage (Ohm et al. 2010). Meanwhile, transcriptome analysis of *Agrocybe aegerita* found that fruiting body stage genes were mainly involved in carbohydrate metabolism and energy production (Wang et al. 2013). Thus, increasing the expression of metabolic pathway genes directly or indirectly provides energy for the formation and development of fruiting bodies. In the present study, we found that 443 (71.57%) of the 605 CAZyme genes were upregulated from the mycelial to the newly differentiated young fruiting body stage (Fig. 2a, b). Further studies showed that the carbohydrate degradation enzymes exo-β-1,4-glucanase, endo-β-1,4-glucanase and β-glucosidase were significantly increased in the fruiting body stage (Fig. 3a, b), and the relative expression of the six CAZyme genes also increased at the mRNA level (Fig. 4a). *M. importuna* is saprophytic and can degrade cellulose, hemicellulose, starch and lignin and use available carbon sources by secreting extracellular enzymes (Zhang et al. 2009; Liu et al. 2017). Carbon sources are based on nutrients for the growth and development of edible fungi, such as *Pleurotus eryngii*, *P. ostreatus and P. pulmonarius* (Stajić et al. 2006). The results suggest that carbohydrate catabolism might provide necessary energy for the formation and development of fruiting bodies in *M. importuna*.

Energy metabolism was also one of the categories with DEG enrichment. In fungi, research has shown that mitochondria produce the majority of cellular energy in the form of ATP and maintain the potential to proliferate and delay cell death (Waterhouse 2003). Additionally, the mitochondrial complex activity levels are proportional to the amount of ATP produced (Dong et al. 2013). In plants, previous research has shown that several mitochondrial proteins play an important role in the regulation of tomato fruit development and quality (Xu et al. 2012). However, the role of mitochondrial proteins in the growth and development of ascomycetes remains unknown. The analysis of the whole transcriptome found that 67 of the 605 DEGs were identified as related to mitochondria, and 49 upregulated DEGs were identified in the *M. importuna* fruiting body formation stage (Fig. 2c). Meanwhile, we found that mitochondrial respiratory chain enzyme activity (complexes I, II, and III) was significantly increased in the fruiting body formation stage (Fig. 3c). In addition, the mRNA expression of 2 mitochondrial ATP synthase genes was proved to be consistent with the activity of the mitochondrial respiratory chain enzyme (Fig. 4b). These results suggest that energy metabolism is significantly enhanced in the formation of the fruiting body and that mitochondrial proteins might be involved in the regulation of the formation of fruiting bodies. Moreover, these results also provide a reference for the study of the role of mitochondrial proteins in the growth and development of ascomycetes.

The fruiting body formation stages of many mushrooms are mediated by cellular processes and genetic, physiological and environmental factors, such as *Schizophyllum commune* (Sen et al. 2016), *Flammulina velutipes* (Ando et al. 2001; Joh et al. 2009), *Coprinopsis cinerea* (Chi et al. 2013), *Boletus edulis* (Zheng et al. 2007) and *Agaricus bisporus* (Ospina-Giraldo et al. 2000; Colauto et al. 2016). Previous studies have shown that increases in the expression of environmentally induced genes, such as *Noxs* (Mu et al. 2014), *dst* (Terashima et al. 2005; Kuratani et al. 2010), *Ubc2* (Zhang et al. 2015), *eln2* (Muraguchi and Kamada 2000); CAZyme genes (Xie et al. 2018); and MAPK, cAMP and ROS signaling genes (Nakajima et al. 2012; Zhang et al. 2015), can regulate the growth and development of edible fungi. Because the environmental factors of *M. importuna* cultivation are difficult to control under natural conditions, these environmental factors might also affect the formation of fruiting bodies. For example, the range of temperature variations from the *M. importuna* mycelial stage to the fruiting body formation stage is 2–12 °C (Liu et al. 2017), diurnal temperature variations of > 10 °C stimulate primordium differentiation, and fruiting bodies cannot grow well at temperatures > 20 °C (Liu et al. 2017, 2018a). Therefore, the temperature increase from the mycelial stage to the fruiting body stage can be considered “heat stress”. However, this “heat stress” is beneficial to the formation and growth of fruiting bodies in the appropriate temperature range. Under heat stress, HSP (heat shock protein) metabolic pathways were found in *Pyropia yezoensis* and in higher plants (Sun et al. 2015). In addition, fungal organisms express HSPs or chaperons to perform biological functions; research has shown that *hsp_60, hsp_90, hsp_104, hsp_30*, and *hsp_10* are expressed as a result of heat stress (Tiwari et al. 2015). In the present study, we found that 19 DEGs encoding HSPs were upregulated in the fruiting body formation stage (Fig. 2c). Further validation by qRT-PCR showed that the expression levels of *hsp_90* and *hsp_12* were significantly increased (Fig. 4b). In basidiomycetes, after *Ganoderma lucidum* mycelium was exposed to heat stress, the expression levels of *hsp_90* and *hsp_70* increased significantly (Tan et al. 2018), which is consistent with our results. In addition, 48 DEGs were also found to regulate carbohydrate metabolism after *Ganoderma lucidum* underwent heat stress (Tan et al. 2018). These results suggest that HSPs may be involved in the formation of fruiting bodies by responding to changes in growth temperature. Meanwhile, changes in growth temperature may also lead to changes in other metabolic pathways, but whether these changes promote or inhibit growth remains unknown.

In addition, changes in growth-related environmental factors may also cause changes in the redox system of *M. importuna*. Previous studies have shown that sclerotium formation in *M. importuna* can be induced by 20 mM H_2_O_2_, and SOD gene expression is also increased (Buscot 1993; Liu et al. 2018b). SOD not only removes the toxicity of active oxygen but also plays an important role in the response of living organisms to stress and cell differentiation (Yan et al. 2016). In plants, studies have found that CAT is also a key antioxidant enzyme against high temperature stress (Almeselmani et al. 2006). In this study, we found that most oxidoreductase DEGs were upregulated in the fruiting body formation stage, and the SOD and CAT genes also showed increased expression levels (Figs. 2c and 3d). Meanwhile, the enzyme activity levels revealed similar variation trends to the gene expression levels (Fig. 4b). The main function of antioxidant enzymes is to remove reactive oxygen species (O_2_^−^ and H_2_O_2_) and reduce the damage to cells under different stresses (Zhang et al. 2017). Therefore, these findings suggest that changes in temperature and other unidentified environmental factors might be involved in the induction of fruiting body formation and might also play an important role in resisting adverse environments.

In conclusion, the mycelium and young fruiting body of *M. importuna* were investigated by transcriptome sequencing, de novo transcriptome assembly, functional annotation and enrichment analysis, resulting in the identification of 51,389 transcripts and 33,269 unigenes, with an average unigene length of 1140.08 bp and an N50 length of 1868 bp. Moreover, 30,477 (59.31%) ORFs were predicted, and 52.55% of unigenes were annotated. A total of 12,561 significant DEGs were identified by transcript RPKM value analysis. The DEGs were mainly enriched in carbohydrate metabolism, energy metabolism and oxidoreductase activity, and HSPs. The enzyme activity assay results indicated that the activity levels of most CAZymes and oxidoreductases were increased in the young fruiting body stage and that the activity of the mitochondrial complex (I, II, and III) in ATP synthesis was also enhanced. Additionally, the expression of genes encoding CAZymes, mitochondrial proteins, oxidoreductases and HSPs was validated by qRT-PCR, and the results revealed trends similar to those observed in the RNA-Seq results. The overall results suggest that the anabolism of carbohydrates and energy provides nutrients for the formation of fruiting bodies, while changes in environmental factors might play an important role in regulating fruiting body formation. In addition, these data provide resources for future studies on achieving high yield and stable production of *M. importuna.*

## Additional file

**Additional file 1: Table S1**. Gene-specific primers used for qRT-PCR. **Table S2.** Number of open reading frames (ORFs). **Table S3.** Top 10 species distribution by unique species hits under NR annotation. **Table S4.** GO functional classification of DEGs. **Figure S1.** Histogram presentation of COG classification. **Figure S2.** GO functional classification of the whole transcriptome of *M. importuna.* **Figure S3.** Histogram presentation of KEGG pathway classification. **Figure S4.** GO functional enrichment analysis of the three clusters of DEGs. **Figure S5.** KEGG functional enrichment analysis of the top 30 significant DEGs.

## Data Availability

The data supporting the conclusions are presented in the main article.

## Abbreviations

NR: nonredundant protein database
GO: Gene Ontology function database
COG: Cluster of Orthologous Groups database
KEGG: Kyoto Encyclopedia of Genes and Genomes pathway database
SWSS: Swiss-Prot protein database
DEG: differentially expressed gene
CAZyme: Carbohydrate-Active Enzymes
qRT-PCR: quantitative real-time PCR
AAs: auxiliary activities
GHs: glycosyl hydrolases
CBMs: carbohydrate binding modules
CEs: carbohydrate esterases
GTs: glycosyl transferases
PLs: polysaccharide lyases
SOD: superoxide dismutase
CAT: catalase
Mito1: ATP synthase
Mito2: mitochondrial F1F0 ATP synthase
HSP: heat shock protein
