# Supplementary material for: Comparative transcriptome analysis reveals potential fruiting body formation mechanisms in Morchella importuna
Source: AMB Express. 2019 Jul 12;9:103. doi: 10.1186/s13568-019-0831-4 (PMC6626090; doi:10.1186/s13568-019-0831-4)
Supplement: Supplementary file 1 — Additional file 1: Table S1. Gene-specific primers used for qRT-PCR. Table S2. Number of open reading frames (ORFs). Table S3. Top 10 species distribution by unique species hits under NR annotation. Table S4. GO functional classification of DEGs. Figure S1. Histogram presentation of COG classification. Figure S2. GO functional classification of the whole transcriptome of M. importuna. Figure S3. Histogram presentation of KEGG pathway classification. Figure S4. GO functional enrichment analysis of the three clusters of DEGs. Figure S5. KEGG functional enrichment analysis of the top 30 significant DEGs. [file 13568_2019_831_MOESM1_ESM.docx]

**Additional file**

Figure S1 Histogram presentation of COG classification

Figure S2 GO functional classification of the whole transcriptome of *M. importuna.*

Figure S3 Histogram presentation of KEGG pathway classification

Figure S4 GO functional enrichment analysis of the three clusters of DEGs.

Figure S5 KEGG functional enrichment analysis of the top 30 significant DEGs.

Table S1 Gene-specific primers used for qRT-PCR

Table S2 Number of open reading frames (ORFs)

Table S3 Top 10 species distribution by unique species hits under NR annotation

Table S4 GO functional classification of DEGs

Figure S1 Histogram presentation of COG classification


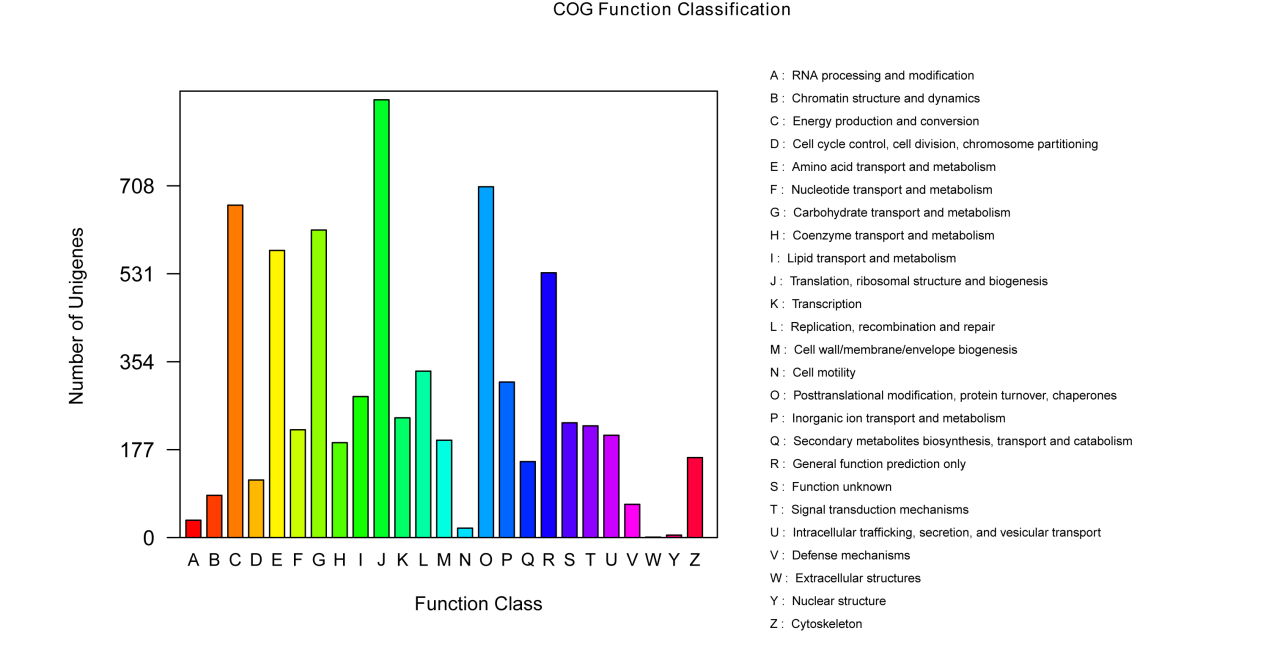


Figure S2 GO functional classification of the whole transcriptome of *M. importuna.*


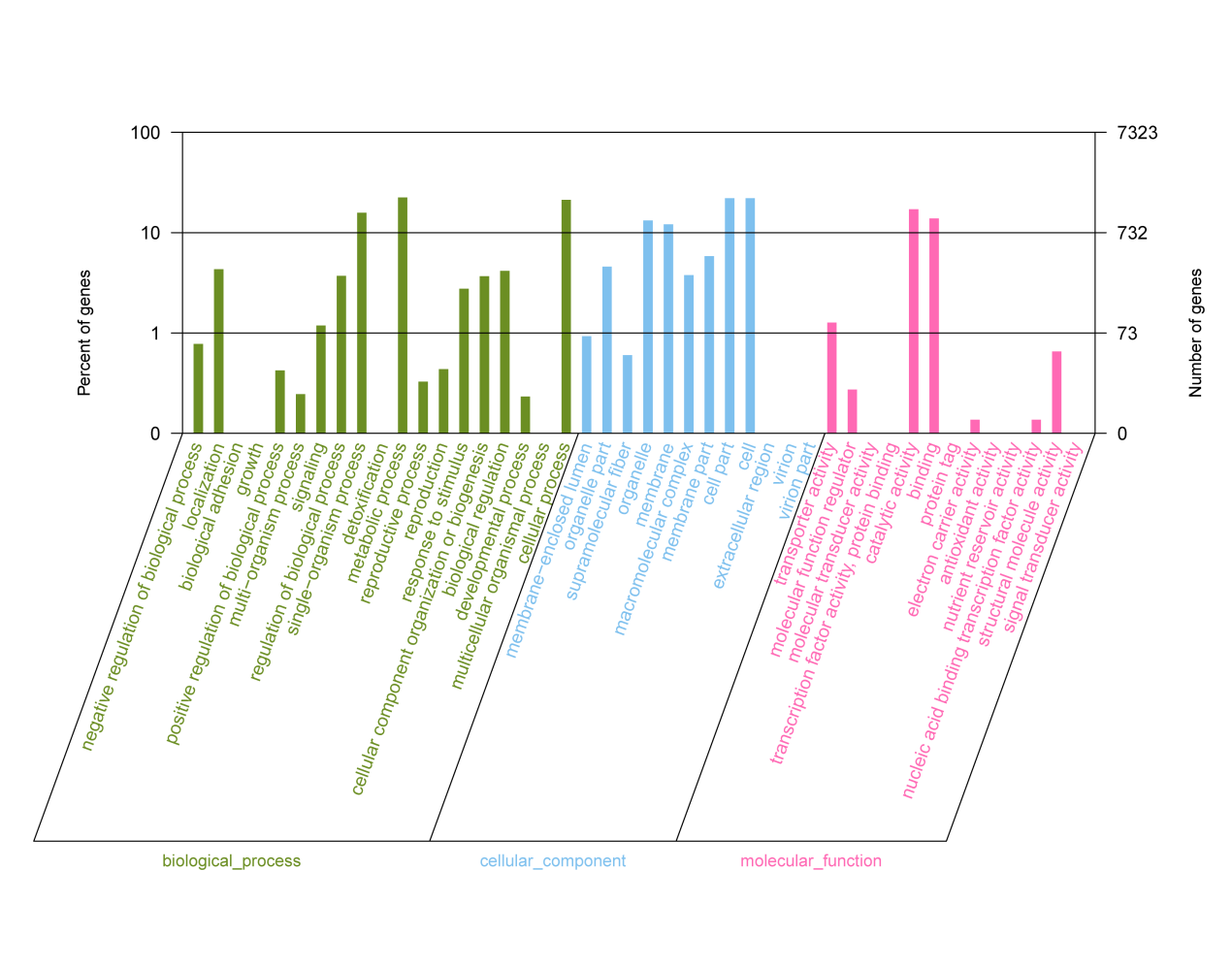


Figure S3 Histogram presentation of KEGG pathway classification.

the KEGG pathways mainly containing divided into five branches:

A: Cellular Processes; B:Environment Information Processing; C:Genetic Information Processing; D:Metabolism; E: Organismal Systems


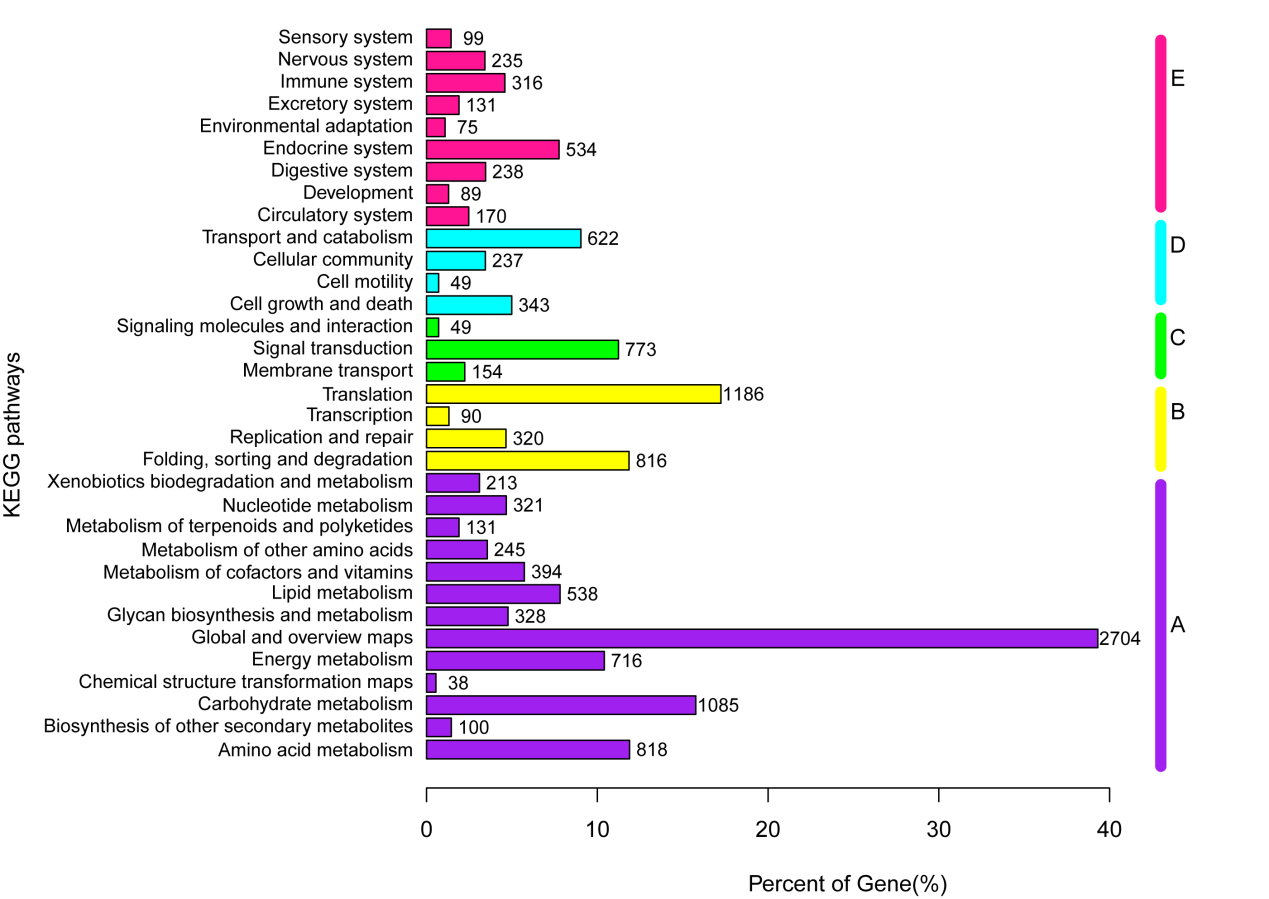


Figure S4 GO functional enrichment analysis of the three clusters of DEGs.





Figure S5 KEGG functional enrichment analysis of the top 30 significant DEGs.


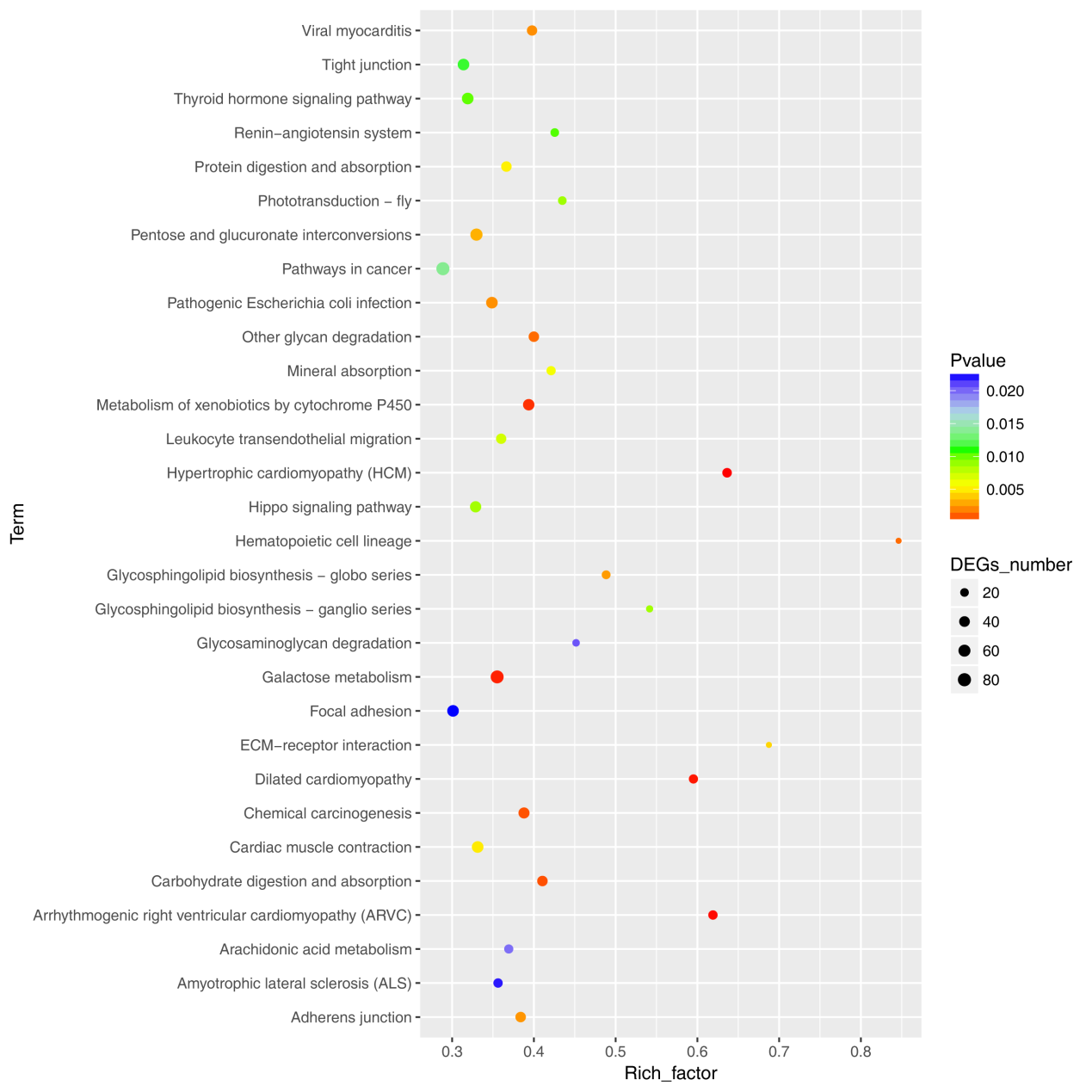


Table S1 Gene-specific primers used for qRT-PCR

| **Primer Name** | **Sequence 5'→3'** | **Accession Number** |
| --- | --- | --- |
| 14-3-3 F | ACCTTGCCGAGTTCGCTAC | KYQ43010.1 |
| 14-3-3 R | GACCGAGACGGATGGGAT |  |
| AA7 F | CAGTACACGACGAATAAGGT | XP_018661925.1 |
| AA7 R | GGCTCGGAGGAGACAA |  |
| GH5 F | GTTGCGGCATACTCGG | CCX07214.1 |
| GH5 R | TGGCATATCGCTGGGT |  |
| PL1 F | TACTCACCTCAACACTCAA | KIY68468.1 |
| PL1 R | AACACCGATGTCCTTTA |  |
| GT2F | GGTATAGACAGATAGGCA | XP_002836325.1 |
| GT2R | ACCGACGATTACTTGA |  |
| CMB1F | CGAGACGGTAGGGATA | KZV73344.1 |
| CMB1R | GGCTTCAACGACAAAC |  |
| CE10F | GTGAGGCTTATGACGG | OWY45439.1 |
| CE10R | TTGACCAGAAAGATAGTTG |  |
| Mito1F | TCTGGAAACTTGACCC | OBZ83218.1 |
| Mito1R | TACGAGCAGGATGGAA |  |
| Mito2F | CCGCCGTTTCGCATCCACTG | EPQ64357.1 |
| Mito2R | CGTTCTGCCTCCAACCTTCAT |  |
| SOD F | GGACTTGCACCCAACACCAA | ADM26044.1 |
| SODR | CGCCATTCGCATCAGATAGA |  |
| CATF | CTCGGCTGATACTGTTCGTG | KXN73085.1 |
| CATR | GTGTAGCTGGATTCCGTTTT |  |
| HSP12F | TCATAAAGACAACAATACA | XP_001268300.1 |
| HSP12R | GAGAAGGATGCTCACA |  |
| HSP90F | CTACGAGAAGAAGCCGAAAG | CCX12261.1 |
| HSP90R | TTGAGACCACGAAGAGCATT |  |

Table S2 Number of open reading frames (ORFs)

| **name** | **Total ORFS** | **Predicted ORFS** | **Unpredicted ORFS** | **predicted ratio** |
| --- | --- | --- | --- | --- |
| Mycelium | 21335 | 12006 | 9329 | 56.27% |
| Fruiting body | 30051 | 18471 | 11580 | 61.46% |
| Tatol | 51386 | 30477 | 20909 | 59.31% |

Table S3 Top 10 species distribution by unique species hits under NR annotation

| **Species** | **Hit number** |
| --- | --- |
| *Tuber aestivum* | 3172 |
| *Tuber melanosporum Mel28* | 2663 |
| *Pyronema omphalodes CBS 100304* | 717 |
| *Beauveria bassiana D1-5* | 673 |
| *Basidiobolus meristosporus CBS 931.73* | 236 |
| *Spizellomyces punctatus DAOM BR117* | 193 |
| *Rhizophagus irregularis DAOM 181602* | 131 |
| *Nectria haematococca mpVI 77-13-4* | 97 |
| *Gonapodya prolifera JEL478* | 94 |
| *Neonectria ditissima* | 91 |

Table S4 GO functional classification of DEGs

| The three categories | The thirty-nine subcategories | up-regulated  gene unmber | down-regulated  gene unmber | The proportion of up-regulated genes |
| --- | --- | --- | --- | --- |
| biological_process | reproduction | 27 | 7 | 79.41% |
|  | metabolic process | 1000 | 439 | 69.49% |
|  | cellular process | 1011 | 424 | 70.45% |
|  | reproductive process | 20 | 4 | 83.33% |
|  | signaling | 50 | 19 | 72.46% |
|  | multicellular organismal process | 2 | 1 | 66.67% |
|  | developmental process | 31 | 10 | 75.61% |
|  | growth | 16 | 3 | 84.21% |
|  | single-organism process | 689 | 379 | 64.51% |
|  | response to stimulus | 134 | 42 | 76.14% |
|  | localization | 286 | 123 | 69.93% |
|  | multi-organism process | 24 | 6 | 80.00% |
|  | biological regulation | 235 | 79 | 74.84% |
|  | cellular component organization or biogenesis | 215 | 61 | 77.90% |
|  | locomotion | 1 | 0 | 100.00% |
|  | biological adhesion | 3 | 0 | 100.00% |
|  | biological phase | 2 | 0 | 100.00% |
| cellular_component | extracellular region | 19 | 4 | 82.61% |
|  | cell | 909 | 261 | 77.69% |
|  | membrane | 401 | 280 | 58.88% |
|  | membrane-enclosed lumen | 92 | 14 | 86.79% |
|  | macromolecular complex | 407 | 63 | 86.60% |
|  | organelle | 637 | 172 | 78.74% |
|  | organelle part | 344 | 78 | 81.52% |
|  | membrane part | 241 | 188 | 56.18% |
|  | cell part | 887 | 260 | 77.33% |
| molecular_function | nucleic acid binding transcription factor activity | 5 | 8 | 38.46% |
|  | catalytic activity | 709 | 368 | 65.83% |
|  | structural molecule activity | 9 | 1 | 90.00% |
|  | transporter activity | 103 | 59 | 63.58% |
|  | binding | 736 | 279 | 72.51% |
|  | electron carrier activity | 7 | 4 | 63.64% |
|  | antioxidant activity | 7 | 3 | 70.00% |
|  | enzyme regulator activity | 20 | 4 | 83.33% |
|  | protein binding transcription factor activity | 4 | 0 | 100% |
|  | molecular transducer activity | 1 | 5 | 16.67% |
|  | molecular function regulator | 20 | 5 | 80.00% |
|  | protein tag | 3 | 0 | 100% |
|  | nutrient reservoir activity | 0 | 1 | 0 |
|  | translation regulator activity | 0 | 1 | 0 |
